# The Grieving Process of a Family Caregiver: Experience Before Influences What Happens Next—A Grounded Theory

**DOI:** 10.3390/nursrep16060201

**Published:** 2026-06-12

**Authors:** Catarina Simões, Margarida Vieira, Ana Paula Sapeta

**Affiliations:** 1 Department of Nursing, Santa Maria Health School, Travessa Antero de Quental, 173, 4049-024 Porto, Portugal; 2 CIIS Centro de Investigação Interdisciplinar em Saúde, Universidade Católica Portuguesa, 4169-005 Porto, Portugal; 3 RISE-Health, Santa Maria Health School, Travessa Antero de Quental, 173, 4049-024 Porto, Portugal; 4 Dr. Lopes Dias High School of Health, Castelo Branco Polytechnic Institute, 6000-084 Castelo Branco, Portugal; 5 AGE.COMM Unidade de Investigação Interdisciplinar—Comunidades Envelhecidas Funcionais, Castelo Branco Polytechnic Institute, 6000-084 Castelo Branco, Portugal

**Keywords:** bereavement, grief, family caregiver, nursing care, grounded theory

## Abstract

**Background/Objectives**: Recognizing and managing grief is particularly important in nursing, especially from the perspective of family caregivers. In this qualitative study, we aim to understand the grieving process of family caregivers, focusing on what happens before the death of an adult family member due to chronic illness, and to identify the factors influencing the grieving process in this context. **Methods**: This study is an outcome of a broader study which aimed to understand how family caregivers grieve during the first year following the death of an adult family member due to a chronic illness. This article will only address the influencing conditions that emerged from data related to events that occurred prior to the person’s death. A theoretical sample was gathered through semi-structured interviews with 20 bereaved family caregivers. Data were collected and then analyzed independently by the research team using the three stages and principles of Strauss and Corbin’s grounded theory. **Results**: Adaptation was identified as the central category. Before death, the family caregiver undergoes two adaptive processes: adapting to their new role and preparing for the imminent loss. As they adapt to this loss, they become aware of the seriousness of the illness and the inevitability of death, opening the possibility for the grieving process to begin. The process is influenced by personal and contextual factors as well as interaction-related factors, including access to information, satisfaction with the care provided, recognition of their efforts, and feelings of abandonment or interaction with healthcare professionals. A wide range of emotions and feelings are experienced. This experience is colored by hope and anticipatory grief. The meaning of the dying process is explored and expectations are redefined. **Conclusions**: The grieving process experienced by family caregivers is an adaptive process that begins before the patient’s death. Some conditions can be modified before the patient’s death; in this case, nurse interventions can enhance the experience of family caregivers.

## 1. Introduction

Grief is a phenomenon that has been studied in the social sciences, particularly in psychology. This has produced knowledge that is highly relevant to other scientific areas, particularly nursing. Due to the nature of their role, nurses follow up with individuals throughout the life cycle at various levels of prevention. When caring for an adult with a chronic, progressive disease that leads to dependence on others for self-care, whether at home or in an institutional setting, and whether there is a family caregiver present, the nurse has a duty to provide care to the patient/family caregiver simultaneously. However, due to the demands of the tasks involved, when a person with a chronic, progressive disease experiences the end of life at home with a family caregiver, the latter may find it difficult to fulfill both their role as family members and as caregiver. This can affect their preparation for loss and their experience of grief [1].

Grief is one of the most universal human responses, which can be experienced multiple times throughout life, across all cultures [2,3,4]. Nevertheless, each instance of grief is unique and cannot be repeated [5]. The fact that other disciplines have shown interest in this phenomenon means that specific contributions can be gathered from multiple disciplines, enriching knowledge on the subject [6,7] and contributing to a better approach to the phenomenon. Sigmund Freud’s studies are generally considered the starting point for research on grief; although Freud was not directly interested in grief itself, he studied the melancholia or depression resulting from it [7]. As a process, grief has been analyzed and organized into stages, phases, and tasks. The phased approach to grief is essentially associated with the Kübler-Ross psychosocial model; in the domain of cognitive models, Parkes, Bowlby, Sanders, and others have studied grief as a process that unfolds in stages. Worden proposes a set of four tasks in his conceptual model of grief, arguing that this is a more facilitating approach in the context of clinical practice [8]; unlike previous approaches that refer to the passivity of the bereaved person, a task-based approach involves the person carrying out actions aimed at processing the suffering caused by the loss.

Despite the significant progress made in the field of loss and grief research, originally initiated by Freud, prevailing models have failed to address all the issues that arise during the grieving process [7]. Most research in this area is conducted within the field of psychology and primarily focuses on pathological forms of grief, rarely integrating research from other disciplines. Some models, such as the one proposed by Tonkin [9], have received little attention. This model addresses the underestimation of resilience and advocates growth beyond grief, acknowledging the possibility that grief does not necessarily reach a point of “resolution”. Furthermore, existential suffering related to the loss of a significant person has not been properly recognized or sufficiently integrated into dominant models. Guldin and Leget [7] were motivated to propose an integrated model of loss and grief considering these arguments. The Integrated Grief Process Model, influenced by the principles and philosophy of palliative care, argues that patients and their families should be accompanied throughout the entire illness process and that their suffering should be recognized throughout this journey. It proposes, therefore, that grief be perceived similarly to the phenomenon of total pain. From this perspective, the model recognizes physical, psychological, social, and spiritual sources of suffering, in the sense that grief, similar to pain, should not be viewed in a one-dimensional way. The psychological aspects are divided into emotional and cognitive, and all dimensions are interrelated. The spiritual dimension is related to the ability to integrate loss and discover a way to trust and love again. Each dimension is influenced by stress-generating factors and involves the completion of tasks [7].

The medicalization of grief seems to reflect the inadequate training of healthcare professionals in addressing bereavement [10]. As a cross-cutting phenomenon, grief deserves a public health approach [11,12,13]. This requires not only the involvement of health services but also that of the entire community, including local authorities, social services, volunteers, and compassionate communities. The media itself should increase awareness of how to support people in their final days and during bereavement, while also promoting and disseminating artistic and cultural initiatives related to these issues. This would contribute to improved literacy about dying and grief [13].

Research into grief trajectories and the factors that influence them is still a priority in grief research [4].

## 2. Materials and Methods

This study was conducted within a qualitative framework and is one outcome of a broader study that aims to understand how family caregivers grieve during the first year following the death of an adult family member due to a chronic illness. One of the specific objectives was to identify factors that interfere with the grieving process in the year following the death of an adult family member with a chronic illness. This article will only address the influencing conditions that emerged from data relating to events that occurred before the person died. The authors aimed to expand knowledge of this phenomenon and generate empirical findings to help nurses understand the bereavement process experienced by family caregivers in the year following the death of a chronically ill adult patient. When researchers wish to gain a deeper understanding of psychosocial processes and develop a theory to explain what is happening in their area of interest, grounded theory, which is significantly influenced by symbolic interactionism, is an appropriate approach [14,15]. The grounded theory methodology was used, adopting the approach of Strauss and Corbin [14,15]. It was important to consider researchers’ theoretical sensitivity to respond with detail and meaning; their professional and personal experiences and knowledge about the phenomenon were sought in the literature. Interviews were recorded, and observations were documented. The audio files were saved on a password-protected computer accessible only to the principal researcher. Verbatim interview transcripts were created in encrypted Word documents, and the NVivo^®^ software version 1.6.1 was used for data analysis and coding.

### 2.1. Study Population

The study population consisted of bereaved family caregivers who lost a loved one within the last year, those who provided care for a chronically ill adult patient, and who agreed to participate in the study. Only family caregivers over 18 years of age who were registered in community health centers or with a deceased relative under the care of the Department of Internal Medicine at a private hospital in the north of Portugal were included. Participants were identified and referred by healthcare professionals (doctors and nurses) who had been bereaved for different lengths of time, from 0 to 12 months after the death of their relative. The researcher’s initial objective was to verify diversity in the time frame between death and data collection, as well as in sociodemographic characteristics such as gender, age, and family bond.

Two interviews were conducted as pre-tests and were included in the total data set, as no alterations to the content or form of the interview were required. After the theoretical sample reached saturation, two more interviews were conducted to ensure that no new attributes would emerge in the already-identified categories. This resulted in a total of twenty participants: 15 females and 5 males. Regarding family ties, fourteen children and six spouses were interviewed. The referring professionals were informed about the nature of the study and were sensitive and receptive to the requests of the research team; for instance, insofar as it was necessary, participants with specific characteristics were identified in order to enrich the comparative analysis process.

The interviews took place during the first year after the death of the patient, between 3 weeks and 1 year, with 9 of the interviews occurring in the first six months.

The hospital referred eleven participants, while community health functional units referred nine, including the Community Palliative Care Support Team (3), Family Health Unit (4), and Community Continued Care Unit (2). No caregivers were found to be at risk of Prolonged Grief Disorder; however, they would have been excluded if identified.

### 2.2. Data Collection

Theoretical sampling is a data collection process for generating theory, whereby analysts collect, code, and analyze data, deciding which data to collect next and where to find it, in order to develop their theory as it emerges [16]. Semi-structured in-depth interviews consist of an unstructured interview characterized by flexibility and the intention to explore various dimensions of the interviewee’s lived experience. Only one consistent question was asked: “How have you been experiencing this phase of your life now that your relative has passed away?” The interviews were transcribed verbatim by the first author. Records of observations made during data collection were registered. Field notes and diagramming were written and used during the comparative analysis process throughout the study. The interviews took place between October 2023 and April 2024 and were conducted either in the participants’ homes or in an office designated for this purpose at the referring health unit. The average duration of each interview was 88.4 min, with a standard deviation of 2.6 in a total of 1768 min.

During the open coding process, more than half (65%) of the codes referred to the family caregivers experience before patient’s death.

A total of 2436 codes were identified, and a timeline was created during the axial coding process (before, during and after death).

Once theoretical saturation was reached, two more interviews were conducted to ensure that no new attributes would emerge in the categories that had already been identified.

### 2.3. Ethical Considerations

From an ethical perspective, the main issues to be addressed in a study developed using the grounded theory methodology are informed consent, confidentiality and management of any sensitive information to which the researcher has access [16]. Although the study population can be defined as vulnerable, positive effects of bereaved individuals participating in research may include the possibility of sharing their narrative and finding meaning in the loss of a family member [17]. While revisiting the experience may be considered traumatic, it is important to note that the researcher possesses communication skills fostered by her clinical experience in supporting patients and families in palliative care, including bereavement support. These skills helped to create a safe environment and ensure compassionate and respectful responses during the interviews.

Necessary ethical and methodological rigor was observed; the North Regional Health Administration and the hospital’s Ethics Committee favorably considered the requests for opinion (CE/2022/57 in 20 April 2022, HLA-1.2024 in 12 February 2024). Participation in the study was voluntary, and participants were given the option to refuse or withdraw from the study at any time. They consented to be identified by the referring healthcare professional and to participate in the data collection process. To this end, an informed consent document was prepared, which included a summary of the research project to safeguard the rights of each participant.

Data confidentiality and anonymization (through coding) were strictly guaranteed within the context of the study. Only the research team had access to the interview recordings, verbatim transcripts, and memos, which were all stored in a secure cloud folder.

Verifying validity, reliability, and credibility criteria is a crucial requirement when developing a research study, whether quantitative or qualitative. In a qualitative study, the researcher ensures adherence to these criteria throughout the entire process, from data collection, categorization, structuring, and comparison with the existing literature to theorization. To ensure this, the authors considered Strauss and Corbin’s (2015) nine key empirical evaluation criteria designed to assess the rigor and validity of a grounded theory study [18]. Conceptual labels were systematically derived from the raw data and used to form a cohesive, integrated theoretical framework. The main categories were developed in terms of their properties and dimensions, and variations were accounted for through different conditions and consequences. Categories were developed with density, considering conceptual detail and relationships. Processes and context were taken into account, and, as previously stated, theoretical sampling was used to guide data collection and explore evolving concepts [18].

## 3. Results

During axial coding, the relationships between subcategories were examined and articulated. This allowed concepts to be integrated into higher-order categories, contributing to the emergence of a coherent, data-driven explanatory structure. Authors extracted four categories from the pre-death phase that are related to the specific aim of this study, which is to identify the factors that influence the grieving process of family caregivers of adult family members with chronic illnesses. As the sample coding progressed, it followed the outline of the family caregiver’s story, and it has been possible to recognize a set of categories: unavoidable operational actions and strategies, influencing conditions (personal, contextual and related to the interaction), a reinterpretation of experience and experiences and feelings.

During the interviews, when participants were asked how they felt in the present moment, they started to talk about what had happened before the death. The adaptive process was defined as the central category. It emerged before conducting selective coding, since it was possible to identify early in the interviews that family caregivers were adapting over time. The research team also realized that the family caregiver’s adaptive process in response to the death of an adult with a chronic illness occurred at three distinct stages: before death, during the final days and hours of life (or imminent death), and after death.

**Figure 1:**
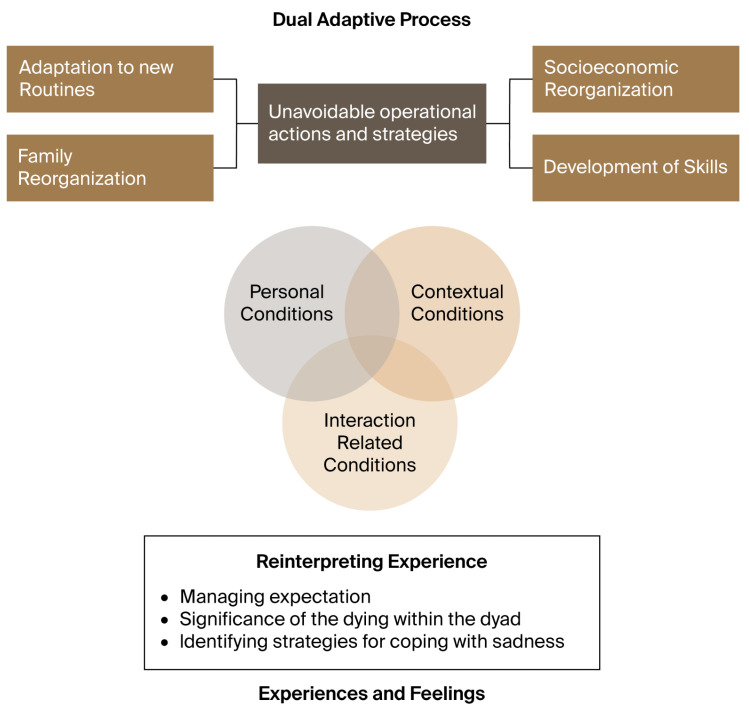
Grieving process of family caregiver before death of family member.

Before the patient dies, the family caregiver experiences a dual adaptive process: adapting to their new role and preparing for the imminent loss of their family member. This adaptive process is represented in Figure 1.

The process begins when the family caregiver has access to information regarding the serious illness of their family member. Data analysis revealed that after this moment, operational actions and strategies inevitably take place. The adaptive process is influenced by personal, unmodifiable conditions related to the caregiver’s own personality, modes of self-care, and emotional bond with the patient. In addition to personal factors, the bereavement process experienced by a family carer of an adult with a chronic illness is also influenced by contextual factors and by conditions related to the interaction continuum.

### 3.1. Essential Actions and Strategies for Adaptation

During the grieving process, family caregivers develop essential adaptation strategies. Family and socioeconomic reorganization, adaptation to new routines and the development of new skills emerged as subcategories. During the process of adapting to the role of family caregiver, he seeks to reorganize their life, whether from a family or socioeconomic perspective. In addition to providing care for the person in their charge, the family caregiver continues to perform their professional duties and other tasks associated with their role within the family, such as caring for other family members or ensuring that household chores are completed. Family reorganization is made easier with family support, although the family caregiver often wishes to avoid overburdening other family members.

*It was very important that I could take turns with my sister, so that we could get some rest, because the days and nights with my mother were very difficult.*

(I 17)

Difficulties in providing care are usually associated with a lack of knowledge and resources, as well as deficient social support. The family caregiver adapts to their new role by adjusting their availability to fit in the new tasks. Sometimes, introducing new routines requires suspending others, such as physical activity. Although this is valued by the family caregiver, it can be perceived as less relevant. The new routine may also entail moving to a new physical space, such as moving in with the patient or receiving him/her in one’s own home. Family caregivers develop new skills associated with their role and as bereaved family members. These skills correspond to the operational actions and strategies that are unavoidable during the grieving process of a family caregiver of an adult with a serious chronic illness.

### 3.2. Personal Conditions

The adaptive process is influenced by personal, unmodifiable conditions related to the caregiver’s own personality, adherence to their own self-care routines and emotional bond with the patient.

*What gave me strength while I was caring for and accompanying him? I am a serene person, and I have a lot of patience. I have always been an early childhood education teacher.*

(I 12)

*Did you see the film Love? It’s amazing, but the way the two of them shut themselves off and put their daughter away can only end badly. I think there’s a need to maintain contact with the outside world and a connection.*

(I 16)

*I knew my husband very well. We started dating when we were sixteen and have lived together ever since. I think that helped us a lot when it came to understanding what was left unsaid.*

(I 12)

### 3.3. Contextual Conditions

Seven categories related to contextual conditions were extracted. They may be related to work activity, the availability of a formal caregiver, the perception of safety in the care setting, the characteristics of the dying process, the burden of tasks, the complexity of care, and the possibility of being present.

Family caregivers with active professional ties may find it difficult to meet work demands, feeling the need to reduce their workload or associated responsibilities, or at least have flexible hours that allow them to care for the patient, for example by giving them the possibility of working remotely.

*I initially asked to work remotely, but that request was denied. However, I had scheduled holiday time a long time ago, starting on the 19th, and she passed away on the 26th, so the last seven days were already within my holiday period. Before that, I was always working.*

(I 17)

The availability of a formal caregiver allows the family caregiver to maintain their work activity and creates space for them to preserve their self-care. This formal caregiver may be someone referred to and hired for this purpose, or someone from the family caregiver’s circle who already performs domestic tasks.

*I had a lot of support from an employee of mine who lives in the house with me, my housekeeper. She doesn’t have children either, she’s very dedicated. She was always by my side, helping us.*

(I 10)

During the care process, the family caregiver, either in a home or hospital setting, needs and values safety. The need for a secure environment is often understood by the family caregiver as a crucial reason for keeping the patient at home and seeking a hospital setting only when they do not feel safe providing care at home.

*From the moment they told me that, if necessary, everything would come here (to his home) and that he would receive oxygen if he needed it, I felt more at ease.*

(I 4)

The way in which a family caregiver deals with their grief is affected by the features of the patient’s dying process, notably the perception of a path of suffering. The inability to communicate with the person, witnessing their cognitive and physical deterioration, the presence of difficult-to-control signs and symptoms, and the way the person experiences the pathological process of dying and its duration are characteristics of the dying process that influence the grieving process of the family caregiver. The idea of a peaceful death, one that is seemingly free from suffering, can evoke feelings of gratitude and tranquility.

*When she passed away, I felt grateful for the serene and peaceful way she left, and for the way she was treated in hospital.*

(I 19)

Barriers to communication with the patient, stemming from cognitive decline, prostration, or periods of confusion, are identified as hindering the family caregiver’s adaptation process, as they impede the exchange of relevant messages associated with reconciliation to the dying process. The family caregiver develops new communication strategies, paying attention to nonverbal communication and developing new communication codes.

*He stopped talking—or rather, his eyes did—but he kept moving further away.*

(I 16)

An overload of tasks emerges as a condition that hinders the grieving process, either because it prevents self-care or it reduces the time that can be shared with the rest of the family (spouse or children).

*I had worked two nights in a row that week. They weren’t consecutive, but I never recovered. I was always being called in.*

(I 4)

The complexity of caregiving also presents itself as a challenging condition, requiring the development of specific skills for which the family caregiver may not feel prepared. Caring for a person with cognitive impairments who exhibits aggressive behavior can be very demanding. It requires a learning process aimed at developing skills, which can be challenging for family caregivers.

*She would come home at night and curse at me in ways I had never heard my mother speak. It wasn’t her; it was latent anger. Then she turned to me as an outlet. I said, ‘I can’t take it anymore.’*

(I 19)

Family caregivers feel that their presence is important and that this facilitates the grieving process by providing reassurance. In the home setting, there are generally no constraints on this, except when the family caregiver maintains a professional activity, relying on the help of another family member or a formal caregiver to provide care. In a hospital setting, presence is usually managed by healthcare professionals, according to the institution’s guidelines, but also based on an assessment of the situation.

*I don’t think I would have coped well if I hadn’t had the opportunity to be with my mother. In my mother’s case, being present certainly made things much easier.*

(I 17)

The family caregiver values the fact that the healthcare professional (doctor or nurse) is flexible with visiting hours and/or allows them to accompany the patient during procedures, including in the emergency room.

### 3.4. Conditions That Emerge in the Interaction Continuum

Interactions with the healthcare team influence the adaptive process experienced by the family caregiver before the death of the patient (Table 1). Both facilitating and hindering conditions were identified. The grieving process of the family caregiver is influenced by information, in terms of communication patterns and the provision of sufficient and intelligible information. Regarding the intention of the healthcare team, the experience can be facilitative.

*“This will happen, that will happen…” It’s a whirlwind, but I have a map to guide me. I found that very useful at the time and still do now.*

(I 6)

Understanding the patient care plan will depend on how the information is conveyed, both in terms of the communication style used and the intelligibility of the discourse. Knowledge of the expected clinical course, included in an individual and integrated care plan that is understood, discussed, and in which the family caregiver feels involved, facilitates the grieving process, creating the conditions needed for their necessary awareness and adaptation to the role of family caregiver. In the absence of a clear communication process, this leaves room for ambiguous messages and the dubious interpretation of their content. As a result, the family caregiver attempts to extract meaning from the healthcare professional’s nonverbal communication, which limits their understanding of the situation due to the use of language that is difficult for the interlocutor to understand.

*If they had told me when she went to intensive care… I have some regrets because they didn’t tell me how ill she was. If they had told me, ‘Her condition is serious. She needs to go to intensive care’, I wouldn’t have come home. But they just said, ‘It’s better to take her to intensive care’, without telling me how serious the situation was. I didn’t understand at the time. I didn’t say goodbye. I didn’t realise how serious the situation was.*

(I 14)

In addition to the lack of validation, the transmission of insufficient information hinders the adaptation process, which becomes especially relevant at the time of hospital discharge. In addition to the quantity and quality of information received, the family caregiver is sensitive to the pattern of communication, especially when delivering bad news. The way such information is registered and remembered is proportional to the perceived lack of care from healthcare professionals.

*If I could go back and change that moment, I would. I would say, ‘Maybe we should go to an office where we can talk more calmly.’ Having to deal with that information in a hallway, followed by my brother asking, ‘And when did they tell us that?’, left a mark on us.*

(I 5)

Satisfaction with healthcare influences and facilitates the grieving process for family caregivers. When caregivers feel that the healthcare team listens to and respects the patient–family caregiver dyad, they perceive a commitment to care. Recognition of the family caregiver’s efforts is also a determining element.

*She was so attentive and it was so important that Nurse S came here and listened to me. I kept thinking how important it was…*

(I 8)

The grieving process for family caregivers is facilitated when they feel their efforts are recognized by a team of healthcare professionals.

*I called them here and, by chance, they came. They were impeccable, I can’t deny that. Nurse S came and calmed me down on the phone, telling me how to administer the medication and reassuring me that I was doing everything very well.*

(I 8)

Conversely, the grieving process is negatively affected when their perspective is not considered in the evaluation and discussion of the care plan.

*The nurses realized that he was too unstable to go home. In other words, it was difficult to define exactly what was wrong with him. Instead of asking my opinion, they decided not to try to see if he would calm down if I was there.*

(I 1)

Following on from this, some factors may make the adaptation process difficult for family caregivers. When they feel their efforts are undervalued and face a situation where they perceive they are not receiving the necessary support, family caregivers feel abandoned by the healthcare team.

*Caring for someone without support and without them listening to us made this whole process exhausting for both of us.*

(I 12)

*… What they said was, “You do it like this, cut it, glue it, and you can go home and take care of him” (person with a colostomy). My anger started there; sometimes I felt alone, I felt abandoned…”*

(I 9)

A lack of rapid response to an acute event, particularly when the patient is at home, or the absence of regular follow-up is considered a potentially disruptive factor that endangers the safety of home care and undermines trust in healthcare professionals.

*It was very difficult. She missed her appointments and everything had to be done by phone because nobody could visit her. They had to go by ambulance to see her; no one could come to her house. It was very difficult.*

(I 1)

A lack of support and insensitivity to the suffering of the patient, a lack of coordination among nurses within the same team, and between interdisciplinary team members, hinder the adaptation process.

*There were many moments when she had finally managed to fall asleep and endure the pain, only for someone to burst in and ask what she wanted for breakfast. Then, five minutes later, they would ask what she wanted for lunch. Then, another five minutes later, they would ask what she wanted for a snack. And then, yet again, they would ask what she wanted for dinner. Couldn’t they have asked all of that at the same time?*

(I 2)

*And then, in the middle of it all, even after she had managed to rest, the nurse would come to change the IV, change her position or administer pill A or B. I realized there was a huge lack of coordination, and above all an inability to understand that they could reduce those moments to just one…*

(I 1)

Perceiving a lack of support from the healthcare team, or a lack of specialized care for oneself or the patient, hinders the adaptation process and creates the perception of a lack of access to quality care.

*The hospital never showed any concern either. They didn’t even call after he was discharged, despite how ill he was. They didn’t even send an email to ask if we had received any support.*

(I 3)

A lack of information about nursing care and a lack of training for family caregivers are perceived as obstacles to adaptation.

*The nurses went there too, but we didn’t understand what they were giving him or doing. They didn’t explain anything.*

(I 1)

*I wanted her to explain to me, or to both of us, “this is natural, it’s possible that this or that might still happen.*

(I 2)

### 3.5. Experiences and Feelings

For some family caregivers, the caregiving process is an act of love, representing an opportunity to reciprocate the care given to a parent. Family caregivers are often preoccupied with the patient’s well-being, striving to meet their needs, even if this sometimes involves a significant financial burden. However, this leads to difficulties associated with fatigue, conditioned by the nature of caregiving and reduced time for self-care, as well as difficulties associated with the suffering they witness. This experience can awaken memories of previous losses due to death, which may have been multiple and successive effects.

In this dual adaptive process, hope colors the caregiver experience and is reconfigured over time. The family caregiver introduces changes associated with a reality that is anticipated as real and imminent, namely, the death of the patient and living with anticipatory grief.

Upon becoming aware of the inevitability of death due to a chronic pathological process, the family caregiver begins to rationalize, feel informed, and try to prepare for the person’s absence. Throughout this experience, the family caregiver copes, with varying degrees of difficulty, with changes in sleep patterns and appetite, often associated with weight loss. Despite the physical manifestations mentioned, some family caregivers avoid the use of medication, fearing that its use will reduce their ability to adequately care for the sick person.

### 3.6. Reinterpreting Experience

The awareness process that emerges from the fact that the dyad, composed of the patient and the family caregiver, is informed about the expected clinical course, leads to the re-signification of the dying process between the two individuals. It often awakens a reflection on life, the appreciation of feelings, a strengthening of the bond, and the integration of death as a natural, inevitable, and ever-present process in their lives.

*But death started to become real, and we started to face it. We were already having conversations and saying goodbye a little every day. Death became a presence. For me, for her…*

(I 11)

In this sense, starting from the reinterpretation of the dying process between the dyad, the possibility of progressively more open communication is created, which facilitates the management of realistic expectations, based on an honest approach to the main fears and concerns of everyone. As mentioned previously, being informed about the pathological process and the expected clinical course of the family member are facilitating factors. This allows the dyad to focus on the present moment, experience it intensely and meaningfully, and try to maintain hope of living longer. Alternatively, it allows moments of consented crying and the development of strategies to cope with sadness.

## 4. Discussion

The experience of being a family caregiver before the death of a loved one is remembered after their death and is considered a key factor in discussions about grief. In the year following the death of an adult with a chronic illness, the bereaved family caregiver recalls memories related to the moment of diagnosis of the serious chronic illness, or the recognition of its deterioration, either by themselves or by a healthcare professional. They feel that their experience of the entire process leading up to the death influences how they cope with the bereavement.

During the grieving process, the family caregiver acts and develops strategies that are essential for adaptation: family and socioeconomic reorganization, adapting to new routines, and developing new skills. During the process of adapting to their role as family caregivers, they seek to reorganize their life, whether from a family or socioeconomic perspective [19,20]. This requires the expenditure on significant temporal and financial resources. Introducing new routines requires the suspension of others, such as physical activity. The new routine may also entail moving into the patient’s home or receiving him/her in their own place. Family caregivers also develop new skills associated with caregiving and bereavement after reaching a turning point that triggers the need to fulfil the role of both a carer and a bereaved family member [21].

The way in which family caregivers of adults with chronic illnesses adapt to the grieving process is influenced by personal, contextual and emergent conditions within the continuum of interactions. Those related to the interaction continuum have the greatest potential for modification, with the aim of improving the experience of the family caregiver and avoiding additional suffering. Throughout the care process, the family caregiver comes to understand how healthcare professionals interact with each other and recognizes the benefit of interdisciplinary teamwork, valuing the fact that the nurse coordinates with the rest of the nursing team, as well as other team members. Information is crucial and understanding symptoms and knowing how to intervene are both protective factors [22]. The effectiveness of the care plan depends on how information is communicated, in terms of both the style of communication and the clarity of discourse. Familiarity with the expected clinical course, as set out in an individualized care plan that has been discussed and in which the family caregiver feels involved, facilitates the grieving process by creating the conditions necessary for awareness and adaptation to their role. This plan should be developed based on a communication exercise centered on the values of the patient and their family [23].

Monitoring the entire end-of-life process for the patient and their family caregiver is key to determining the grieving process, particularly in terms of managing multidimensional symptoms to prevent and alleviate suffering. The characteristics of the dying process and the complexity of care itself influence the adaptive process. Family caregivers value interdisciplinary teamwork, which is characterized by true collaboration between all stakeholders, placing the patient and their family at the center of the care plan. Support for family members can involve empowering them in their role as caregivers. This facilitates the presence of family members in the context of hospitalization and recognizes and values their efforts in the care process. Finally, communication is essential to verify all the conditions that influence the adaptive process of grief. Transmitting information intelligibly, progressively, sensitively, and articulately among the various actors in the care plan promotes adequate expectation management and ensures the awareness process necessary for adaptation.

Limitations of this study include the fact that, of the 22 people who were referred to and eligible to participate in the study group, two declined, stating that they did not feel capable of taking part in a study of this nature. It was also not possible to collect data from the parents of adult children or siblings, which could have provided a broader perspective and made the theory more applicable to the phenomenon under study.

### Implications for Clinical Practice

Throughout the life cycle, the family nurse is well-positioned to support individuals and families, addressing grief and facilitating transitions. They can also refer individuals to a specialized team when recognizing signs of complex grief or risk of disturbance. From a public health perspective, primary prevention entails adequate monitoring of patients and their families throughout the chronic disease process to prevent and alleviate their suffering.

Reflecting on the results of this study, the implications for nursing can be analyzed from the perspective of clinical practice, teaching, and research. Understanding the grieving process of the bereaved family caregiver, both before and after the patient’s death, allows us to understand that this should be a focus of nurses’ attention. By analyzing the contextual conditions and those that emerge in the continuum of interaction, as well as the best scientific evidence, it is inferred that improving the care provided at end-of-life facilitates the adaptation process of the caregiver/bereaved family member towards grief. The important role of the family caregiver must be recognized, and the adaptation process they undergo must be acknowledged, valued and supported in a systematic and intentional way. The family caregiver should be seen as a member of the team and as a care recipient, who needs to be well in order to continue providing care, but also so that the adaptation process can be experienced in a healthy way and to catalyze a healthy grieving process. When reflecting on the implications of this study’s results from the perspective of nursing education, it is important to ensure adequate training and preparation, and identify more innovative teaching/learning strategies; for instance, simulated practices can be utilized in relation to programmatic content that involves the development of skills in the field of communication. Understanding the grieving process of family caregivers during the first year after the death of an adult relative suffering from a chronic illness will lead to the development of a nursing intervention model in subsequent phases of this research.

## 5. Conclusions

During the grieving process, the family caregiver develops operational strategies that are essential for adaptation, the sharing of experiences and feelings, and the reinterpretation of their own experience. It can be inferred that this process occurs in three distinct phases: before, during, and after the death of the patient.

Given that the grieving process for family caregivers begins before death and is influenced by factors such as the characteristics of the dying process, the complexity of care, the possibility of being present (which is relevant when care does not occur in a home setting), recognition and appreciation of the family caregiver’s role, and access to and quality of information, it is clear that a comprehensive approach must consider these influencing factors to ensure care is provided to both the patient and the family caregiver. This can facilitate a healthy transition to the grieving process.

Understanding the grieving process experienced by the family caregivers of adults with chronic illnesses and applying this knowledge to nursing can improve bereavement care in this context.

Research conducted by nurses should yield implications for clinical practice to improve the effectiveness and quality of nursing care.

## Figures and Tables

**Table 1:** Conditions influencing the interaction continuum.

INTERACTION RELATED INFLUENCING CONDITIONS	
Facilitators	Obstacles
**Information**	Feeling of abandonment
**Satisfaction with healthcare**	Delay in follow-up
**Recognition of the family caregiver’s efforts**	Insensitivity towards pain and suffering
	Lack of overall or specialized support
	Lack of teamwork coordination
	Lack of information about nursing care
	Lack of training for family caregivers

## Data Availability

Data is unavailable due to privacy and ethical restrictions.

## Public Involvement Statement

Bereaved carers were involved in data collection (interviews).

## Guidelines and Standards Statement

This manuscript was written against the Standards for Reporting Qualitative Research.
